# Deep Reinforcement Learning-Based Secrecy Rate Optimization for Simultaneously Transmitting and Reflecting Reconfigurable Intelligent Surface-Assisted Unmanned Aerial Vehicle-Integrated Sensing and Communication Systems

**DOI:** 10.3390/s25051541

**Published:** 2025-03-02

**Authors:** Jianwei Wang, Shuo Chen

**Affiliations:** The Key Laboratory of Modern Measurement and Control Technology, Ministry of Education, Beijing Information Science and Technology University, Beijing 102206, China; chenshuo@bistu.edu.cn

**Keywords:** integrated sensing and communication (ISAC), simultaneously transmitting and reflecting reconfigurable intelligent surface (STAR-RIS), unmanned aerial vehicle (UAV), secrecy, multi-agent deep reinforcement learning (MADRL)

## Abstract

This study investigates security issues in a scenario involving a simultaneously transmitting and reflecting reconfigurable intelligent surface (STAR-RIS)-assisted unmanned aerial vehicle (UAV) with integrated sensing and communication (ISAC) functionality (UAV-ISAC). In this scenario, both legitimate users and eavesdropping users are present, which makes security a crucial concern. Our research goal is to extend the system’s coverage and improve its flexibility through the introduction of STAR-RIS, while ensuring secure transmission rates. To achieve this, we propose a secure transmission scheme through jointly optimizing the UAV-ISAC trajectory, transmit beamforming, and the phase and amplitude adjustments of the STAR-RIS reflective elements. The approach seeks to maximize the average secrecy rate while satisfying communication and sensing performance standards and transmission security constraints. As the considered problem involves coupled variables and is non-convex, it is difficult to solve using traditional optimization methods. To address this issue, we adopt a multi-agent deep reinforcement learning (MADRL) approach, which allows agents to interact with the environment to learn optimal strategies, effectively dealing with complex environments. The simulation results demonstrate that the proposed scheme significantly enhances the system’s average secrecy rate while satisfying communication, sensing, and security constraints.

## 1. Introduction

The rapid development of sixth-generation communication technology (6G) has led to an increasing requirement for intelligent machine-type communication (IMTC), and the integration of sensing technologies to facilitate intelligent communications has emerged as an inevitable trend. Integrated sensing and communication (ISAC) has garnered significant interest from both studies and businesses as an essential research approach for 6G in recent years [1,2,3,4]. By integrating communication and sensing capabilities, ISAC can markedly improve spectral efficiency, reduce system complexity, and offer technical support for developing application contexts such as smart cities and intelligent transportation.

Compared to traditional terrestrial communication, the use of unmanned aerial vehicles (UAVs) as ISAC base stations (UAV-ISAC) offers significant advantages, particularly in providing line-of-sight links and optimizing the quality of communications. UAV-ISACs leverage their mobility to dynamically adjust flight altitudes and trajectories to avoid obstructions, enhancing the stability of communication and sensing connections while flexibly adapting to diverse environments. Furthermore, the three-dimensional mobility of UAVs allows them to extend their coverage to remote or obstructed areas that terrestrial base stations cannot easily reach, significantly improving overall system coverage. For instance, Meng et al. [5] have highlighted that UAVs can maintain uninterrupted communications while performing sensing tasks. Xu et al. [6] further enhanced this framework through the addition of computing capabilities for real-time data processing and decision-making. Xiao et al. [7] proposed improving the communication rate through dynamic resource allocation and beamforming to enhance obstruction resistance. These studies underscore how UAVs can strengthen the flexibility and expand the coverage of communications, driving the advancement of UAV-assisted ISAC systems.

While UAVs can adapt to different scenarios through adjusting their trajectories, factors such as obstacles, line-of-sight variations, and user distribution uncertainties still limit their communication-related performance in complex urban environments. Therefore, reconfigurable intelligent surfaces (RISs) have been introduced to enhance wireless systems. Pan et al. [8] have provided a comprehensive overview of recent advancements in RIS-aided wireless systems from a signal processing perspective, highlighting promising research directions that warrant further exploration. Accurate channel state information (CSI) estimation is crucial for practical RIS systems. In this aspect, Zhou et al. [9] have proposed a novel cascaded channel estimation strategy for RIS-assisted multi-user MISO systems, addressing the high overhead caused by the passive nature of RISs and the large number of reflecting elements. Byun et al. [10] have introduced a two-stage channel estimation technique for UAV-RIS communication systems, estimating them at different time scales. In this study, in order to simplify the system design, we assume the use of perfect CSI.

RISs have been widely used to enhance the stability and reliability of communications in UAV-ISAC systems. Wu et al. [11] have proposed an RIS-assisted UAV-enabled ISAC system that optimizes the trade-off between communication and sensing through jointly designing the RIS phase shift, UAV trajectory, beamforming, and user scheduling, as well as an iterative optimization algorithm for improved performance. Conventional RIS configurations require the transmitter and receiver to be positioned on the same side, significantly restricting the flexibility of their deployment and limiting system coverage. To overcome these limitations, simultaneously transmitting and reflecting reconfigurable intelligent surfaces (STAR-RISs) enable the independent control of both transmission and reflection (T&R) channels, allowing signals to serve users on both sides of the surface. This dual functionality not only enhances system flexibility but also significantly expands communication and sensing coverage, making STAR-RISs particularly advantageous in complex and dynamic environments [12]. Chen et al. [13] have proposed a STAR-RIS-assisted DFRC-UAV-enabled ISAC system that maximizes the multicast capacity through jointly optimizing scheduling, transmission covariance, RIS coefficients, and the UAV trajectory, using an alternating optimization algorithm for sub-optimal solutions.

In the ISAC scenario, security issues are often of significant concern due to the multi-functional nature of integration, which expands the attack range. The system architecture is complex, and dynamic environments increase the risk of vulnerabilities. Yu et al. [14] have studied secure transmission in ISAC via RIS-UAV systems through the introduction of artificial noise interference and jointly optimizing UAV deployment, beamforming, and noise power to enhance the signal quality, thus addressing the non-convex problem. Salem et al. [15] have proposed the use of active RIS to optimize the secrecy rate of MU-MISO in ISAC systems through a jointly designed beamforming approach to enhance physical layer security, with the results demonstrating that active RISs outperforms passive RISs. Although the research presented in [14,15] employed an RIS-assisted ISAC, it was still based on a fixed-base station ISAC architecture. Compared to the UAV-ISAC approach, the transmission distance is more constrained, making it more susceptible to signal attenuation and blockage issues, which may affect the system’s stability and coverage. Zhang et al. [16] have proposed the joint optimization of resource allocation, RIS phase shifts, and the UAV trajectory to enhance the secrecy rate and energy efficiency in integrated sensing and communication scenarios. They employed an RIS-assisted UAV-ISAC scheme to maximize the secrecy rate; however, this scheme utilizes a conventional reflecting-only RIS. Different from traditional RISs, STAR-RISs can simultaneously control the reflection and transmission of signals, providing greater flexibility, making them particularly suitable for application in complex environments. However, research on the security of STAR-RIS-assisted UAV-ISAC systems is still relatively limited, and enhancing the security of such systems remains a crucial area of investigation.

Traditional convex optimization methods are widely used in RIS-assisted ISAC systems; however, their limitations become apparent when dealing with non-convex problems and high-dimensional state–action spaces. Reinforcement learning (RL), with its strong adaptability, has emerged as an essential tool for solving complex problems. In STAR-RIS-assisted UAV-ISAC systems, decision-making problems can be modeled using Markov decision processes (MDPs), further enhancing the effectiveness of RL. However, conventional RL algorithms face issues such as low sample efficiency and inadequate exploration of policies in high-dimensional continuous action spaces. Deep deterministic policy gradient (DDPG), as a deep reinforcement learning (DRL) approach, was designed for continuous action spaces. Moon et al. [17] have used the DDPG algorithm to optimize beamforming at both the ISAC base station and the RIS mounted on a UAV, aiming to maximize the secrecy rate. Additionally, the twin delayed deep deterministic policy gradient (TD3) approach resolves the problem of Q-value overestimation through the use of a dual-network architecture, delayed target updates, and target policy smoothing, serving to reduce Q-value overestimation while improving stability and sample efficiency, making TD3 more reliable for continuous control tasks. In STAR-RIS-assisted UAV-ISAC systems, TD3 can optimize the UAV trajectory, beamforming, and STAR-RIS phase control, effectively handling dynamic environments and improving both the quality and security of communications. Compared to single-agent DRL, multi-agent deep reinforcement learning (MADRL) employs a distributed strategy, decomposing the system into multiple intelligent modules (e.g., STAR-RIS and UAV-ISAC), effectively reducing computational burdens while enhancing system scalability and flexibility. While maintaining efficient learning and stability, MARL effectively addresses complex environments, improving the system’s adaptability and robustness.

In response to the communication performance requirements of UAV-ISAC systems, this study introduces STAR-RIS technology to overcome the limitations of normal RISs in terms of deployment and coverage range. As such, a STAR-RIS-assisted UAV-ISAC system framework is formulated, leveraging the wide coverage capabilities and flexible deployment advantages of both STAR-RISs and UAVs to enhance the overall system performance. To address security issues, the average secrecy rate (ASR) is set as the optimization objective, with the aim of improving the security and anti-eavesdropping capabilities of the communication system. The multi-agent twin delayed deep deterministic policy gradient (MATD3) algorithm is adopted to jointly optimize the UAV trajectory, beamforming, and the amplitude and phase adjustment of the STAR-RIS reflection elements, maximizing the ASR while ensuring that relevant system constraints are satisfied. The contributions of this study can be summarized as follows:

(1) This study introduces a framework for a STAR-RIS-assisted UAV-ISAC system, where the UAV operates in three-dimensional space to provide downlink communication services to legitimate ground users. The framework jointly optimizes the UAV-ISAC trajectory, beamforming, and the phase and amplitude of the STAR-RIS reflection elements with the objective of maximizing the ASR while ensuring that the system meets the SINR constraints for communication, sensing, and eavesdropping.

(2) The MATD3 algorithm is employed to optimize the system’s decision-making process, simultaneously optimizing the UAV-ISAC trajectory, beamforming, and STAR-RIS configuration. This optimization maximizes the ASR of the system, demonstrating the effectiveness of the proposed algorithm.

The remainder of this paper is organized as follows: Section 2 presents the system model. Section 3 focuses on the system model of the STAR-RIS-assisted UAV-ISAC network and the problem of maximizing the ASR. Section 4 describes the simulation results, and Section 5 provides this paper’s conclusion.

## 2. System Model and Problem Formulation

In this section, we establish the system model, introduce the STAR-RIS-assisted UAV-ISAC system, and formulate the problem of maximizing the ASR.

### 2.1. System Model

**Figure 1:**
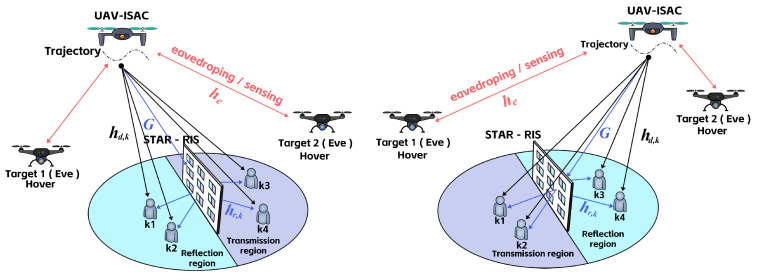
STAR-RIS-assisted UAV-ISAC system.

We consider a STAR-RIS-assisted UAV-ISAC system, as shown in Figure 1. In this scenario, legitimate communication users are distributed freely on both sides of the STAR-RIS. Meanwhile, some UAVs act as eavesdroppers, hovering around the area to intercept the data of legitimate users. The UAV-ISAC controls the trajectory and beamforming to provide downlink communication to legitimate users. The UAV-ISAC is equipped with a uniform linear array (ULA) antenna, consisting of $N_{t}$ antennas. Simultaneously, the UAV-ISAC system uses its sensing capabilities to detect the positions of the eavesdropping UAVs. As shown in Figure 1, Eve represents the eavesdropping UAVs. The STAR-RIS has *M* reflection elements and uses the energy-splitting (ES) protocol [16]. Each STAR-RIS reflection element splits the incoming signal into transmitted and reflected components. The reflection elements of the STAR-RIS control the amplitude and phase, enabling the effective transmission of communications. Each legitimate ground user and eavesdropping UAV is equipped with a single antenna [17,18,19].

We assume that the UAV-ISAC system transmits a set of joint signals containing both communication and sensing signals. This set of joint signals can be expressed as

(1) $$x=W_{c}s_{c}+W_{r}s_{r},$$

where $s_{c}\in C^{K\times l}$ represents the communication symbols for the *K* legitimate ground users, $W_{c}\in C^{N_{t}\times K}$ denotes the corresponding communication beamforming matrix, $s_{r}\in C^{E\times 1}$ denotes the sensing symbols from the UAV-ISAC to the *E* eavesdropping UAV, and $W_{r}\in C^{N_{t}\times E}$ is the beamforming matrix for sensing. Additionally, for simplicity, we define the overall symbol vector $s≜{\left[s_{c}^{T},s_{r}^{T}\right]}^{T}\in C^{(K+E)\times 1}$ and the beamforming matrix $W≜\left[W_{c},W_{r}\right]\in C^{N_{t}\times \left(K+E\right)}.$

(1) Communication Model: The signal received by the *k*-th legitimate ground user can be expressed as

(2) $$y_{k}=\left(\begin{array}{c} h_{d,k}^{H}+h_{r,k}^{H}\Theta_{T/R}G \end{array}\right)Ws+n_{k},$$

where $h_{d,k}\in C^{N_{t}\times 1}$ represents the direct channel from the UAV-ISAC to the *k*-th legitimate ground user, $h_{r,k}\in C^{M\times 1}$ denotes the channel from STAR-RIS to the *k*-th legitimate ground user, $\Theta_{T/R}$ is the STAR-RIS coefficient matrix, $G\in C^{M\times N_{t}}$ is the channel between the UAV-ISAC and STAR-RIS, and $n_{k}∼\mathrm{CN}\left(0,\sigma_{k}^{2}\right)$ represents the zero-mean additive white Gaussian noise (AWGN) at the *k*-th user. In detail, $h_{d,k},h_{r,k},$ and G can be expressed as

(3) $$h_{d,k}=\sqrt{\beta d_{d,k}^{-\alpha }}e_{d,k},$$

(4) $$h_{r,k}=\sqrt{\beta d_{r,k}^{-\kappa }}e_{r,k},$$

(5) $$G=\sqrt{\beta d_{UAV\text{-}ISAC,r}^{-\kappa }}E_{UAV\text{-}ISAC,r},$$

where β is the reference channel power at a distance of 1 m, $d_{d,k}$ represents the communication distance from the UAV-IAC to the legitimate ground user, $d_{r,k}$ represents the distances from the STAR-RIS to the legitimate ground user, and $d_{UAV\text{-}ISAC,r}$ represents the distances from the UAV-ISAC to the sensing target. Additionally, $e_{d,k}$ represents the channel from the UAV-ISAC to the legitimate ground user; $e_{r,k}$ represents the channel from the STAR-RIS to the legitimate ground user; and $E_{UAV\text{-}ISAC,r}$ represents the channel from the UAV-ISAC to the STAR-RIS.

The STAR-RIS adopts the ES protocol. For each STAR-RIS element, the incident signal is divided into transmitted and reflected signals. Therefore, the transmission and reflection factors can be expressed as $\beta_{T,m}e^{j\theta_{T,m}}$ and $\beta_{R,m}e^{j\theta_{R,m}},$ respectively. Furthermore, $\beta_{T/R,m}$ and $\theta_{T/R,m}$ are the amplitude and phase of the STAR-RIS reflection elements, respectively.

Thus, the transmission and reflection coefficient matrix for the STAR-RIS with *M* reflection elements is

(6) $$\Theta_{T/R}=\;diag\left(\left[\sqrt{\beta_{T/R,1}}e^{j\theta_{T/R,1}}\cdots ,\sqrt{\beta_{T/R,M}}e^{j\theta_{T/R,M}}\right]\right).$$

Thus, the received SINR at the *k*-th legitimate ground user can be written as

(7) $${\mathrm{SINR}}_{k}=\frac{{\left|\left(h_{d,k}^{H}+h_{r,k}^{H}\Theta_{T/R}G\right)w_{k}\right|}^{2}}{\sum_{j\neq k}{\left|\left(h_{d,k}^{H}+h_{r,k}^{H}\Theta_{T/R}G\right)w_{j}\right|}^{2}+\sigma_{k}^{2}}.$$

The SINR of the legitimate user is used to express the communication rate of the UAV-ISAC for the *k*-th legitimate user as follows:

(8) $$R_{k}=\log_{2}\left(1+{\mathrm{SINR}}_{k}\right).$$

Therefore, the legitimate ground user communication sum rate (LSR) can be expressed as

(9) $$\mathrm{LSR}=\sum_{k=1}^{K}R_{k}.$$

(2) Sensing model: In the sensing system, we focus on targets with further distance, such as airborne targets, as the main objects of study. Therefore, the propagation distance of the signal reflected by the RIS is much greater than the direct signal transmission distance from the target. The echo signal reflected by the target through the RIS is very weak and contributes almost nothing to the signal-to-noise ratio for target detection and, so, it can be ignored during optimization. Therefore, the transmit beam pattern gain from the UAV-ISAC to the eavesdropping UAV *e* (i.e., the power of the probing signal to the target direction) can be expressed as

(10) $$\Gamma_{r,e}=E\left[|h_{UAV,e}^{H}w_{r,e}{|}^{2}\right]=h_{UAV,e}^{H}w_{r,e}w_{r,e}^{H}h_{UAV,e},$$

where $h_{UAV,e}=\sqrt{\beta d_{UAV,e}^{-K}}e_{UAV,e}$ represents the channel gain for the link between the UAV-ISAC and the *e*-th eavesdropping UAV.

Here, we ignore the interference caused by the reflected signals associated with other UAVs. The eavesdropping UAV can merely be detected using the sensing signals produced by the corresponding UAV-ISAC. To meet the requirements for sensing quality, the beam pattern gain directed at various sensing users must account for the minimum beam pattern gain threshold $\tilde{\Gamma }$ and the echo distance [20]. Then, the following constraint must be satisfied:

(11) $$\Gamma_{r,e}\geq d_{UAV\text{-}ISAC,r}^{2}.\tilde{\Gamma }$$

(3) Security model: The received signal of the *k*-th legitimate user intercepted by the *e*-th eavesdropping UAV can be expressed as

(12) $$y_{e,k}=h_{UAV,e}^{H}w_{k}+n_{e},$$

where $n_{e}∼\mathrm{CN}\left(0,\sigma_{e}^{2}\right)$ represents the AWGN at the *e*-th eavesdropping UAV. The eavesdropping SINR of the *e*-th eavesdropping UAV for the *k*-th legitimate user can be expressed as

(13) $${\mathrm{SINR}}_{e,k}=\frac{{\left|h_{UAV,e}^{H}w_{r,e}\right|}^{2}}{\sum_{j\neq k}^{K+E}{\left|h_{UAV,e}^{H}w_{j}\right|}^{2}+\sigma_{e}^{2}}.$$

Based on the eavesdropping SINR, the communication rate of the *e*-th eavesdropping UAV for the *k*-th legitimate user can be expressed as

(14) $$R_{e,k}=\log_{2}\left(1+{\mathrm{SINR}}_{e,k}\right).$$

The secrecy rate is a widely recognized and crucial security performance metric, as it quantifies the amount of data that can be securely transmitted without being intercepted by eavesdroppers. It plays a vital role in ensuring the confidentiality and privacy of communications in wireless systems. Therefore, the overall system’s secrecy rate is expressed as

(15) $$R_{\mathrm{secrecy}}=\sum_{k=1}^{K}\sum_{e=1}^{E}\left(R_{k}-R_{e,k}\right).$$

Furthermore, the secrecy rate is based on *E* eavesdropping UAVs. Therefore, the ASR can be calculated as

(16) $$\mathrm{ASR}=\frac{1}{E}\sum_{k=1}^{K}\sum_{e=1}^{E}\left(R_{k}-R_{e,k}\right).$$

### 2.2. Problem Formulation

Our objective is to maximize the ASR through jointly optimizing the UAV-ISAC trajectory, the transmit beamforming matrix, and the phase and amplitude of the STAR-RIS, while satisfying the sensing performance requirements and secure transmission constraints for each legitimate user. The optimization problem is formulated as

(17a) $$\begin{array}{cc} P: & \max_{W,\beta_{T/R},\theta_{T,R},x_{UAV},y_{UAV},H_{UAV}}\mathrm{ASR} \end{array}$$

(17b) $$\begin{array}{cc} s.t.\;C1: & \sum_{k=1}^{K+M}{∥w_{k}∥}^{2}\leq P_{\max} \end{array}$$

(17c) $$C2:\Gamma_{r,e}\geq d_{UAV\text{-}ISAC,r}^{2}\tilde{\Gamma }$$

(17d) $$C3:{\mathrm{SINR}}_{k}\geq \Gamma_{k}$$

(17e) $$C4:{\mathrm{SINR}}_{e,k}\leq \Gamma_{e}$$

(17f) $$C5:0\leq \beta_{T,m}\leq 1,\;0\leq \beta_{R,m}\leq 1$$

(17g) $$C6:0\leq \theta_{T,m}\leq 2\pi ,\;0\leq \theta_{R,m}\leq 2\pi$$

(17h) $$C7:0\leq x_{UAV}\leq X_{\max},\;0\leq y_{UAV}\leq Y_{\max}$$

(17i) $$C8:H_{UAV,\min}\leq H_{UAV}\leq H_{UAV,\max},$$

where *W* represents the UAV-ISAC transmit beamforming matrix, including communication and sensing; $\beta_{T/R,m}$ and $\theta_{T/R,m}$ represent the amplitude and phase shift of the STAR-RIS reflection elements, respectively; and $x_{UAV},y_{UAV},H_{UAV}$ denote the components of the UAV trajectory.

Constraint (17b) represents the power constraint, where the transmission power of UAV-ISAC includes the power associated with both communication and sensing. UAV-ISAC needs to satisfy this as the maximum total transmission power. Constraint (17c) ensures that the sensing requirement of each sensing target is guaranteed to meet the minimum threshold. Constraint (17d) represents the minimum SINR of legitimate ground users. This constraint ensures that the UAV-ISAC can provide a certain degree of communication services for each legitimate ground user k. Constraint (17e) represents the maximum eavesdropping value that the eavesdropper *e* receives from legitimate ground user k. Constraint (17f) represents the amplitude optimization constraint for the STAR-RIS, which is divided into reflection and transmission amplitude optimization coefficients. Constraint (17g) represents the phase optimization constraint for the STAR-RIS, which is divided into reflection and transmission phase optimization coefficients. Constraint (17h) and constraint (17i) represent the UAV’s flight area and altitude constraints, respectively.

## 3. The Proposed Algorithm

We define the optimization problem based on maximizing the system’s ASR as an MDP model and propose the STAR-RIS-assisted UAV-ISAC based on the MATD3 algorithm (STAR-RIS-MATD3), which jointly designs the UAV-ISAC trajectory, transmits the beamforming matrix, and adjusts the amplitude and phase of the STAR-RIS. The details of the MDP model and MATD3 are described in the following sections.

### 3.1. Markov Decision Process

We propose a STAR-RIS-assisted UAV-ISAC system. In this system, UAV-ISAC and STAR-RIS operate as independent agents, each autonomously selecting its next action based on the current environmental state. Each agent follows an MDP, which is mathematically defined as a triplet (s,a,r). The state space *s* and action space *a* describe all possible states and actions of the UAV-ISAC and STAR-RIS, while the reward function reflects the reward value *r* associated with taking a specific action in a given state.

Therefore, the state, action, and reward of each agent at time step *t* are described as follows:

(1) State *s*: Considering the time-varying nature of the channel, the state includes the relevant CSI information. Therefore, the state of UAV-ISAC at time *t* is defined as $s_{UAV,t}=\left(x_{UAV}^{\left(t\right)},y_{UAV}^{\left(t\right)},H_{UAV}^{\left(t\right)},G^{\left(t\right)},h_{d,k}^{\left(t\right)}\right).$ The UAV-ISAC state information includes the coordinates of the UAV-ISAC, $G^{\left(t\right)}$ (representing the channel state between the UAV-ISAC and STAR-RIS), and $h_{d,k}^{\left(t\right)}$, which represents the channel state between the UAV-ISAC and the legitimate ground users. The state of STAR-RIS at time *t* is defined as $s_{STAR\text{-}RIS,t}=\left(x_{UAV}^{\left(t\right)},y_{UAV}^{\left(t\right)},H_{UAV}^{\left(t\right)},G^{\left(t\right)}\right),$ where the STAR-RIS state information includes the coordinates of the UAV-ISAC and the channel state between the UAV-ISAC and STAR-RIS.

(2) Action *a*: First, the action of the UAV-ISAC is

(18) $$a_{UAV,t}=\left\{{\left\{w_{k}^{\left(t\right)}\right\}}_{k\in K},{\left\{w_{r,e}^{\left(t\right)}\right\}}_{e\in E},\Delta x_{UAV}^{\left(t\right)},\Delta y_{UAV}^{\left(t\right)},\Delta H_{UAV}^{\left(t\right)}\right\},$$

where $w_{k}^{\left(t\right)}=\beta_{k}^{\left(t\right)}e^{i\theta_{k}^{\left(t\right)}},w_{r,e}^{\left(t\right)}=\beta_{r,e}^{\left(t\right)}e^{j\theta_{r,e}^{\left(t\right)}}$ represent the beamforming expressions for the communication and sensing components of UAV-ISAC, which include the amplitude $\beta_{k}^{\left(t\right)},\beta_{r,e}^{\left(t\right)}$ and phase shift $\theta_{k}^{\left(t\right)},\theta_{r,e}^{\left(t\right)}.$

Furthermore, the STAR-RIS consists of reflection and transmission components, and its action space is defined as

(19) $$a_{\mathrm{RIS},t}=\left\{{\left\{\beta_{T/R,m}^{\left(t\right)}\right\}}_{m\in M},{\left\{\theta_{T/R,m}^{\left(t\right)}\right\}}_{m\in M}\right\}.$$

(3) Reward *r*: The reward aligns with our goal of maximizing the ASR. Therefore, the reward function is defined as

(20) $$r_{t}={\mathrm{ASR}}^{\left(t\right)}=\frac{1}{E}\sum_{k=1}^{K}\sum_{e=1}^{E}\left(R_{k}^{\left(t\right)}-R_{e,k}^{\left(t\right)}\right).$$

### 3.2. ASR Maximization Algorithm Based on MATD3

**Figure 2:**
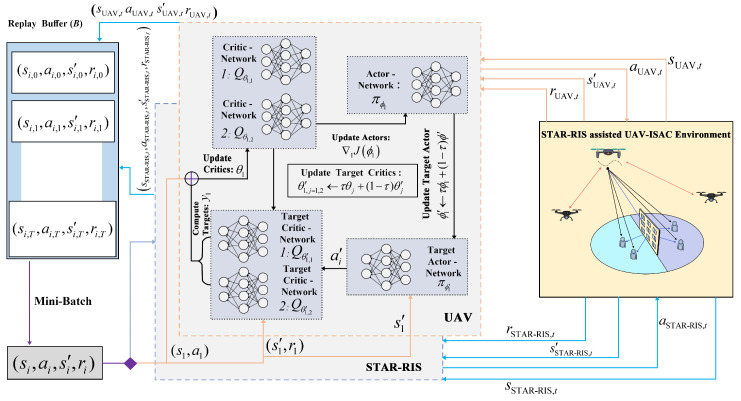
Flow diagram of the MATD3 algorithm.

To address the optimization problem presented in this study, we propose an optimization scheme for maximizing the ASR of the STAR-RIS-assisted UAV-ISAC system based on the MATD3 algorithm, as shown in Figure 2. This algorithm adopts a distributed architecture and incorporates the TD3 algorithm, which avoids the high complexity associated with centralized training and effectively prevents Q-value overestimation, compared to DDPG. To achieve this, we utilized the MATD3 algorithm to jointly optimize the UAV-ISAC trajectory, the transmit antenna’s beamforming matrix, and the amplitude and phase of the STAR-RIS reflection elements, with the goal of maximizing the ASR while satisfying the system constraints. In this approach, the UAV-ISAC and STAR-RIS are modeled as independent agents that interact with the environment through distributed collaboration, gradually learning optimal strategies and further improving the system’s flexibility and efficiency.

Specifically, at each time step *t*, an agent *i* will take an action $a_{i,t}$ based on the current state $s_{i,t}$ using an actor policy network:

(21) $$a_{i,t}\left(s_{i,t}\right)=clip\left(\pi_{\phi_{i}}\left(s_{i,t}\right)+\varepsilon ,a_{\min},a_{\max}\right),\varepsilon ∼N\left(0,\sigma \right),$$

where ε denotes Gaussian noise, and $\alpha_{\max}$ and $\alpha_{\min}$ represent the maximum and minimum values of the action space that the agent can choose, respectively. After the agent takes the action $a_{i,l}$ based on the current state $s_{i,t}$, the agent receives the next state $s_{i,t+1}$ and reward $r_{i,t}$ from the environment. Then, the previous tuple $\left(s_{i,t},a_{i,t},s_{i,t+1}^{\prime },r_{i,t}\right)$ is stored in the replay buffer *B*.

Once enough information is stored in the buffer, the agent will perform mini-batch training by sampling *N* transitions $\left(s_{i,t},a_{i,t},s_{i,t+1}^{\prime },r_{i,t}\right)$ from the buffer. To compute the target value, the agent must use the actor-target policy to select the action $a_{i,t}^{\prime }$ in the next state $s_{i,t}^{\prime }$, and apply clip noise to ensure that the policy is never stagnant by selecting an overestimating action, as shown in Equation (22). Then, Equation (23) is used to calculate the target value $y_{i}$. The agent employs both twin critic-target networks to evaluate the global value of the subsequent state and action, choosing the one with the lowest estimate.

(22) $$a_{i}^{\prime }\left(s_{i}^{\prime }\right)=clip\left(\pi_{\phi_{i}}\left(s_{i}^{\prime }\right)+clip\left(\varepsilon ,-c,c\right),a_{\min},a_{\max}\right),\varepsilon ∼N\left(0,\sigma \right),$$

(23) $$y_{i}=r+\gamma \min_{\theta_{i,j=1,2}}Q_{\theta_{i,j}^{\prime }}\left(s_{i}^{\prime },a_{i,t}^{\prime }\right).$$

Once the agent has its target value $y_{i}$, it will update each of its critic networks $\theta_{i,j=1,2}$ by calculating the mean squared error of these batches of observations and applying a step of gradient descent, as shown in Equation (24).

(24) $$\theta_{i,j}\leftarrow \arg\min_{\theta_{i.j=1,2}}\frac{1}{N}\sum {\left(y_{i}-Q_{\theta_{i,j}}\left(s_{i},a_{i}\right)\right)}^{2}.$$

The agent will update its actor policy $\phi_{i}$ using one of the critic networks at each time step *e*, which is an update interval to estimate the Q-value of the state and the action selected by the policy. Then, we apply a step of gradient ascent to push the parameters of the neural network $\phi_{i}$ in the direction of maximum growth of the Q-value, as shown in Equation (25):

(25) $$\nabla_{\phi_{i}}J\left(\phi_{i}\right)=\frac{1}{N}\sum \nabla_{a_{i}}Q_{\theta_{i,1}}\left(s_{i},a_{i}\right)|a_{i}=\pi_{\phi_{i}}\left(s_{i}\right)\nabla_{\phi_{i}}\pi_{\phi_{i}}\left(s_{i}\right).$$

Once the actor networks are updated, then the target networks are updated using Equation (26):

(26) $$\begin{array}{cc} & \phi_{i}^{\prime }\leftarrow \tau \phi_{i}+\left(1-\tau \right)\phi^{\prime }\\ & \theta_{i,j=1,2}^{\prime }\leftarrow \tau \theta_{i,j}+\left(1-\tau \right)\theta_{i,j}^{\prime }\; \end{array}.$$

Through continuous training cycles until the training period *t* reaches *T*, the UAV-ISAC and STAR-RIS gradually learn the optimal strategy and eventually make decisions that maximize the ASR while meeting the system’s objective requirements. The detailed algorithm is provided in Algorithm 1.
**Algorithm 1:** ASR Maximization Algorithm based on MATD3
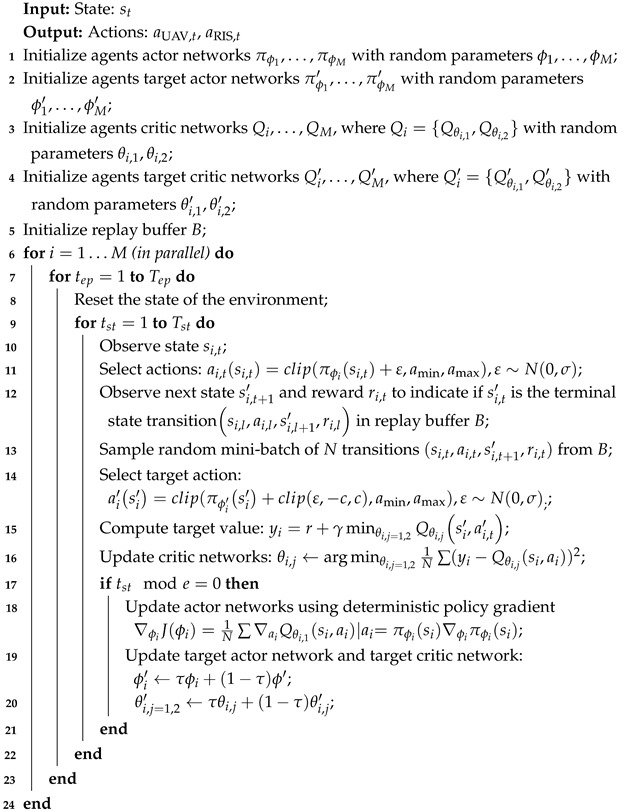


The proposed MATD3 structure consists of the following components. Let *N*, *LA*, and *LB* represent the mini-batch size, the number of layers in the deep neural network (DNN), and the size of each layer, respectively. Additionally, the UAV-ISAC agent has a state space dimension of *a* and an action space dimension of *b*, while the STAR-RIS agent has a state space dimension of *c* and an action space dimension of *d*. We assume a total of $T_{ep}$ episodes, where each agent uses $T_{st}$ steps per episode for training. Therefore, the overall time complexity can be expressed as $O\left(T_{\mathrm{ep}}\cdot T_{\mathrm{st}}\cdot N\left(\left(a+b+c+d\right)LB+LA\cdot LB^{2}\right)\right)$.

## 4. Simulation Results

### 4.1. Simulation Configuration

In this section, we present the simulation results with the aim of validating the performance of the proposed STAR-RIS-assisted UAV-ISAC based on the MATD3 algorithm. We assume a field with an area of 200 × 200 m. The STAR-RIS is located at the center of the region, parallel to the y-axis. The STAR-RIS utilizes the ES protocol and provides communication services to legitimate ground users on both sides through its reflection and transmission functions. Four legitimate ground users are randomly distributed on both sides of the STAR-RIS. Two eavesdropping UAVs are randomly distributed above the region, hovering and intercepting the downlink communication from the UAV-ISAC to the legitimate ground users. The initial position of UAV-ISAC was set to (57,175,60), the final position of UAV-ISAC was set to (60,20,60), and the position of the STAR-RIS is (100,100,20) [21]. Throughout the task, the UAV-ISAC starts at the initial point, moves to the optimal service position for deployment, and returns to the endpoint for charging when its battery is depleted [22]. The simulation parameters are listed in Table 1.

To analyze the performance of the proposed algorithm, we utilized the following algorithms for comparison:

1. **STAR-RIS-assisted UAV-ISAC based on MADDPG (STAR-RIS-MADDPG):** MADDPG is a widely used DRL algorithm designed for continuous action spaces. This comparison algorithm was introduced to highlight the performance advantages of the MATD3 algorithm, particularly in preventing Q-value overestimation [23].
2. **Coupling Relationship of STAR-RIS-assisted UAV-ISAC (CR-STAR-RIS):** The coupled phase and amplitude shift the STAR-RIS-assisted UAV-ISAC system. The coupling relationship between the transmission and reflection coefficients [24] can be defined as follows: $\beta_{T,m}=\sqrt{1-\beta_{R,m}^{2}},\left|\theta_{R,m}-\theta_{T,m}\right|=\frac{\pi }{2}\;\mathrm{or}\;\frac{3\pi }{2}\;.$
3. **Conventional RIS-assisted UAV-ISAC (C-RIS)** [11]: Reflecting-only RISs are deployed to achieve half coverage of the area. This algorithm uses a conventional RIS-assisted UAV-ISAC system to maximize the ASR.
4. **UAV-ISAC without RIS assistance (Without-RIS):** This comparison algorithm does not use an RIS and relies solely on the UAV-ISAC system to maximize the ASR.
5. **STAR-RIS-assisted Ground-ISAC (Ground-ISAC)** [24]: This comparison algorithm uses a STAR-RIS-assisted fixed ground-based station with the ISAC function to serve ground users.

### 4.2. Performance Analysis

**Figure 3:**
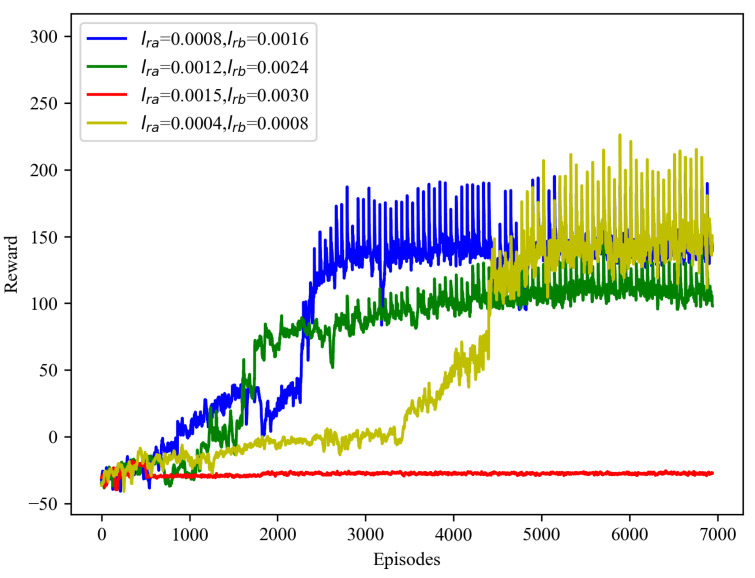
The convergence performance under different learning rates.

Figure 3 illustrates the convergence performance of the STAR-RIS-MATD3 algorithm at different learning rates, with $\left(P_{\max},\;M,\;\tilde{\Gamma }\right)=\left(8\;W,\;15,\;-80\;\mathrm{dBm}\right)$. It can be observed that, when the learning rates are $l_{ra}=0.0015$ and $l_{rb}=0.003$, the algorithm converges too quickly and there is almost no increase in the reward value. This behavior results from excessively large learning rates, leading to large gradient updates that cause the model parameters to overshoot the optimal values, thus hindering effective convergence. On the other hand, when the learning rates are set to $l_{ra}=0.0012$ and $l_{rb}=0.0024$, the reward increases rapidly, but the final converged reward value is lower than the reward when $l_{ra}=0.0008$ and $l_{rb}=0.0016$. This indicates that the algorithm has converged to a local optimum, rather than the global optimum, suggesting that while faster convergence may initially seem beneficial, it may limit overall performance in the long term. In contrast, when the lower learning rates of $l_{ra}=0.0004$ and $l_{rb}=0.0008$ are used, the model learns too slowly, resulting in slower convergence and limiting the model’s ability to adapt to the environment.

To balance efficiency and performance, the learning rates of $l_{ra}=0.0008$ and $l_{rb}=0.0016$ shown in Figure 3 were selected for use in this study. During the first 600 iterations, the algorithm is in the exploration period, and the strategy has not yet matured, resulting in poor performance. When the number of iterations reaches around 2000, the performance of the algorithm is enhanced, and the UAV-ISAC has found an appropriate deployment position to provide services of a certain quality level to the legitimate ground users. Further observation shows that, around the 2800th iteration, the reward value increases again, indicating that the UAV has found a better beamforming strategy, thereby further improving the performance. Eventually, after several iterations, the reward value reaches its optimal level, indicating that the system has found the best deployment solution globally.

**Figure 4:**
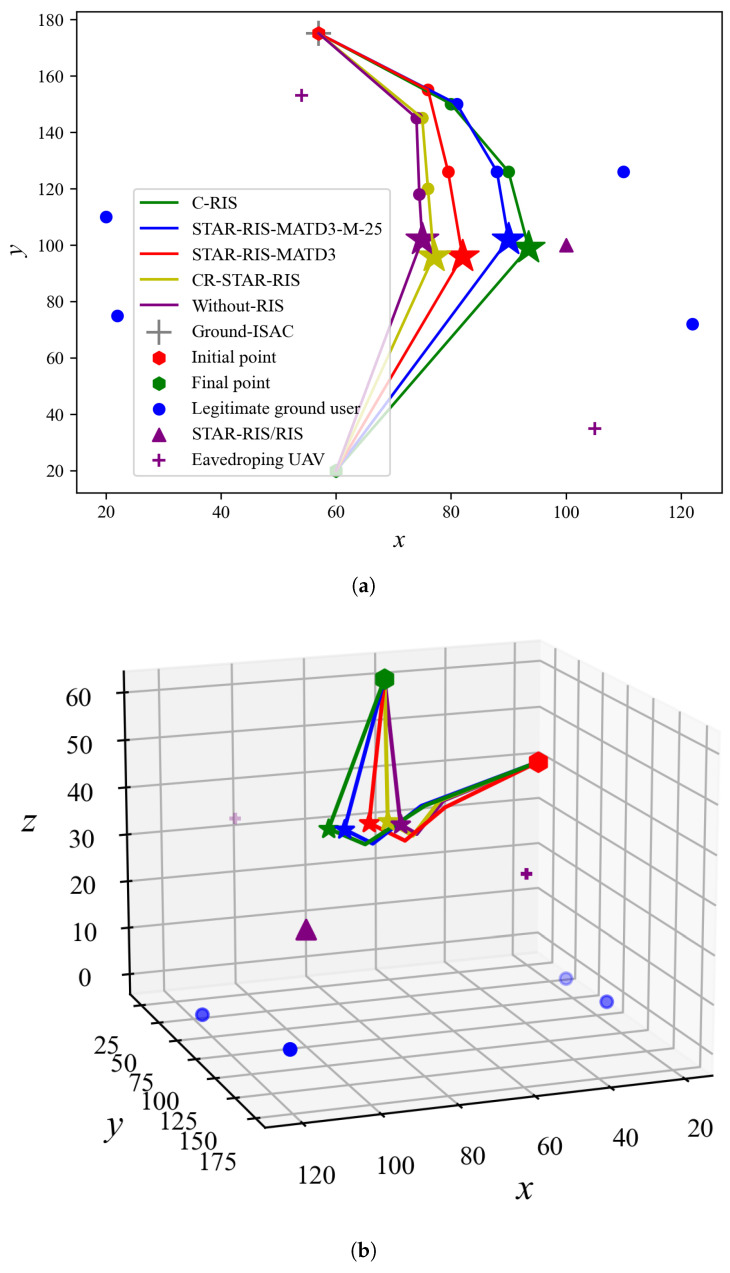
Two- and three-dimensional UAV-ISAC trajectories and optimal hovering positions. (**a**) Two-dimensional UAV-ISAC trajectory and optimal hovering positions. (**b**) Three-dimensional UAV-ISAC trajectory and optimal hovering positions.

Figure 4 shows the UAV-ISAC trajectories and optimal hovering positions under different algorithms in both two- and three-dimensional spaces, with $\left(P_{\max},\;M,\;\tilde{\Gamma }\right)=\left(8\;W,\;15,\;-80\;\mathrm{dBm}\right)$. In Figure 4, the star represents the optimal hover position of different algorithms, while the circle represents the path points of different algorithms to reach the optimal hover position From Figure 4a, we can observe that all UAV-ISAC-based algorithms intentionally avoid eavesdropping UAVs when moving toward the optimal hovering positions. The main reason for this strategy is that maintaining a certain distance helps to reduce the risk of eavesdropping, thus ensuring secure communications. All algorithms chose to hover on the left side of the STAR-RIS/RIS when the UAV-ISAC reached its optimal position. This is because, in order to serve both the left and right legitimate ground users, the UAV-ISAC opts to hover on the left side to take advantage of the shorter direct link between the UAV-ISAC and the legitimate ground users on the left, thereby more effectively improving the performance of the algorithm. As the number of reflection elements *M* in STAR-RIS-MATD3-M-25 increased from 15 to 25, Figure 4 shows that the UAV-ISAC hovering position came closer to STAR-RIS compared to the STAR-RIS-MATD3 algorithm with *M* = 25. This happens as the increased number of reflection elements strengthens the STAR-RIS link, making the UAV more likely to use this link, thereby improving communication performance. For the CP-STAR-RIS algorithm, the coupling strategy of STAR-RIS limits its flexibility, leading to lower reliance on the RIS. As a result, the hovering position is slightly farther from the STAR-RIS compared to STAR-RIS-MATD3. The C-RIS algorithm uses conventional RIS, which only serves users on a single side. To serve users on the back side of the RIS, the optimal hovering position is closer to the RIS. In contrast, the Without-RIS algorithm depends on the positions of legitimate ground users and eavesdropping UAVs. As a result, the UAV-ISAC tends to hover at the center of all legitimate users, which places it to the left of the hovering positions of other algorithms. Additionally, as shown in Figure 4a, when the UAV-ISAC is in the optimal hovering position, its altitude is 40 meters, which is the minimum value of its controllable operational range. This lower altitude reduces the distance to both the legitimate ground users and STAR-RIS, which minimizes path loss and improves communication quality. Finally, after completing the service, the UAV-ISAC flies toward the endpoint.

**Figure 5:**
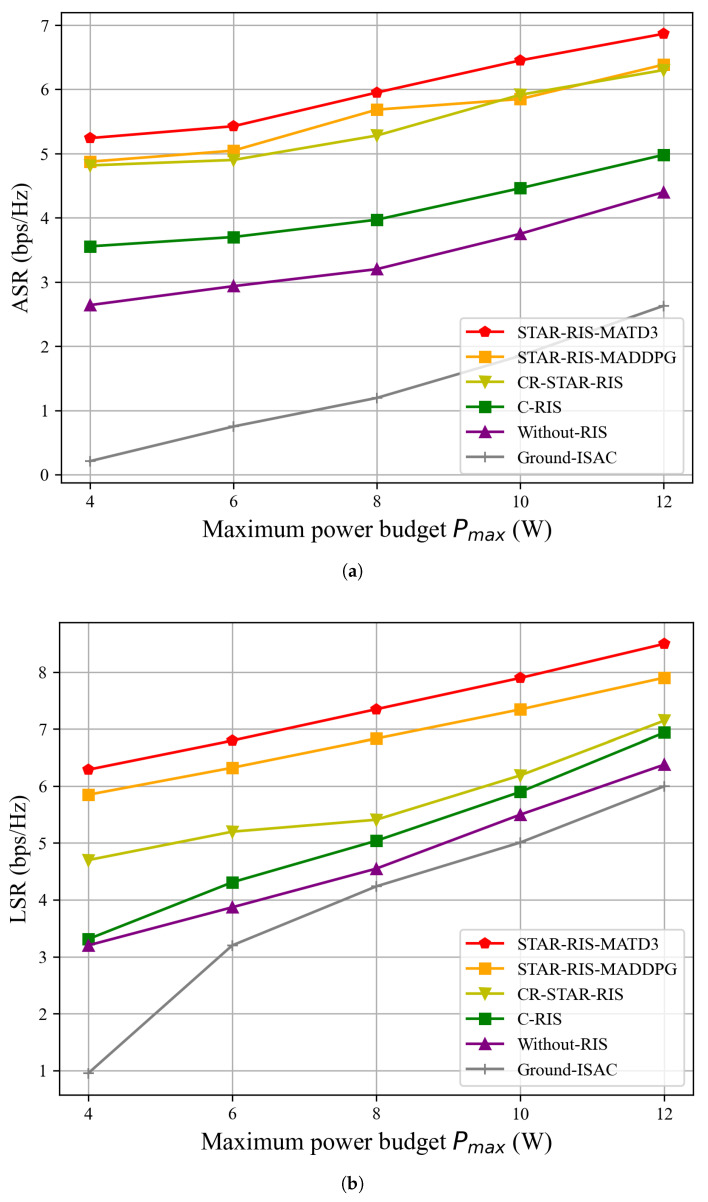
ASR and LSR versus maximum power budget $P_{max}$ for the proposed algorithm and benchmarks, $\left(M,\;\tilde{\Gamma }\right)=\left(15,\;-80\;\mathrm{dBm}\right)$. (**a**) ASR versus maximum power budget $P_{max}$. (**b**) LSR versus maximum power budget $P_{max}$.

Figure 5 shows the relationship between the ASR and LSR under different values of the power budget $P_{max}$, where the system parameters are set to $\left(M,\;\tilde{\Gamma }\right)=\left(15,\;-80\;\mathrm{dBm}\right)$. It can be observed that, as the maximum power budget $P_{max}$ increases, the ASR and LSR of all algorithms present an increasing trend. On one hand, in Figure 5a, increasing the transmission power directly enhances the SINR of each user, thereby improving the signal reception quality. On the other hand, in Figure 5b, in addition to improving the communication quality of legitimate ground users, the increased transmission power not only aids in detecting the locations of eavesdropping UAVs but also effectively interferes with them, reducing the signal reception capability of eavesdropping UAVs. Meanwhile, the ASR is greater than the communication sum rate for all algorithms, except for Ground-ISAC. By leveraging the mobility of the UAV-ISAC, improved secrecy performance can be achieved. The use of the UAV-ISAC allows for adaptive positioning, which enhances the system’s ability to maintain secure communication links. This mobility enables the UAV-ISAC to optimize its trajectory and beamforming strategies, thereby improving the ASR and ensuring more robust protection against eavesdropping.

Importantly, in Figure 5, the proposed algorithm can be seen to have significantly outperformed the other algorithms in terms of the ASR and LSR. This is attributed to its flexibility in controlling the reflection elements of the STAR-RIS provided by our proposed algorithm. Among the comparison algorithms, STAR-RIS-MADDPG, which uses the MADDPG algorithm, suffers from overestimation issues, leading to slightly lower performance when compared to the STAR-RIS-MATD3 algorithm. CR-STAR-RIS uses a coupled strategy for the amplitude and phase of the reflection element matrix, which limits the flexibility of control, thus resulting in lower performance than the STAR-RIS-MATD3 algorithm proposed in this study. The C-RIS algorithm, which relies solely on a reflection function, has a service area which is reduced by half, resulting in a significant decrease in performance. The Without-RIS algorithm depends entirely on the UAV-ISAC for downlink communication to legitimate ground users, leading to significantly lower performance compared to algorithms that use the STAR-RIS or RIS. The Ground-ISAC scheme performed the worst, as it deploys ISAC on a fixed base station without mobility. In this scenario, the eavesdropping UAVs are located between the fixed base station and the eavesdropping UAV, making it easier for a large amount of information to be intercepted, thus leading to the poorest performance.

**Figure 6:**
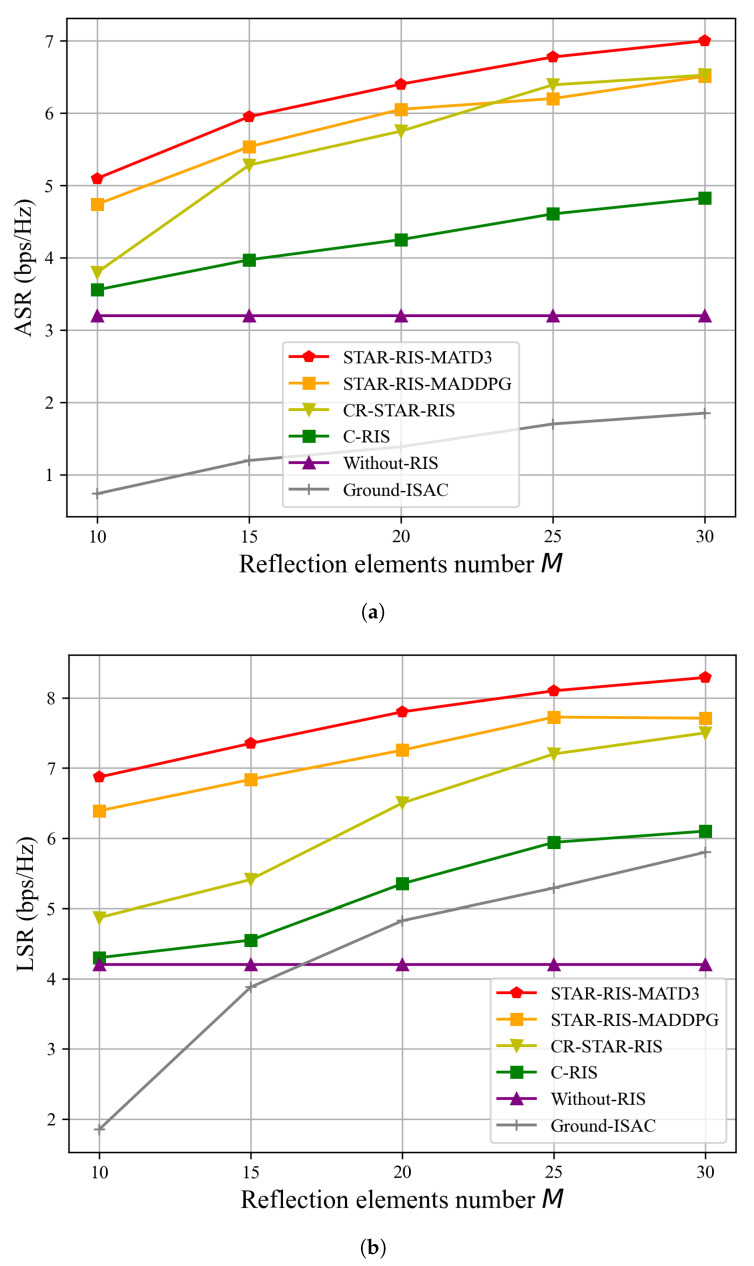
ASR and LSR versus number of reflection elements *M* for the proposed algorithm and benchmarks, with $\left(P_{\max},\;\tilde{\Gamma }\right)=\left(8\;W,\;-80\;\mathrm{dBm}\right)$. (**a**) ASR versus number of reflection elements *M*. (**b**) LSR versus number of reflection elements *M*.

In Figure 6, we evaluate the performance of ASR and LSR while increasing the number of reflection elements *M*, with $\left(P_{\max},\;\tilde{\Gamma }\right)=\left(8\;W,\;-80\mathrm{dBm}\right)$. As shown in Figure 6, increasing the number of reflection elements *M* leads to improvements in the ASR and LSR for all algorithms that utilize reflection elements. This indicates that increasing the number of reflection elements contributes to enhanced security of the system. Figure 6a shows a trend of increasing LSR as the number of reflection elements *M* increases, suggesting that the improvement in LSR is the primary factor contributing to the enhancement of the ASR shown in Figure 6b. In the system design, the STAR-RIS or RIS plays a crucial role, not only enhancing signal reflection or transmission effects but also significantly improving the system’s beamforming capabilities. As the number of reflection elements *M* grows, the system can more precisely adjust the signal’s propagation path, optimizing signal transmission and leading to increases in both ASR and LSR within the system. Moreover, STAR-RIS-MATD3 demonstrates superior security, as it can independently adjust the amplitude and phase of both transmission and reflection, enabling it to simultaneously serve legitimate ground users on both sides. In contrast, CR-STAR-RIS-TD3 has limited flexibility due to the coupling of the transmission and reflection amplitude and phase, while C-RIS-MATD3 can only serve legitimate ground users on one side. Therefore, as shown in Figure 6, STAR-RIS-MATD3 achieved a superior LSR which, in turn, enhances its ASR performance, ultimately improving the overall security of the system.

**Figure 7:**
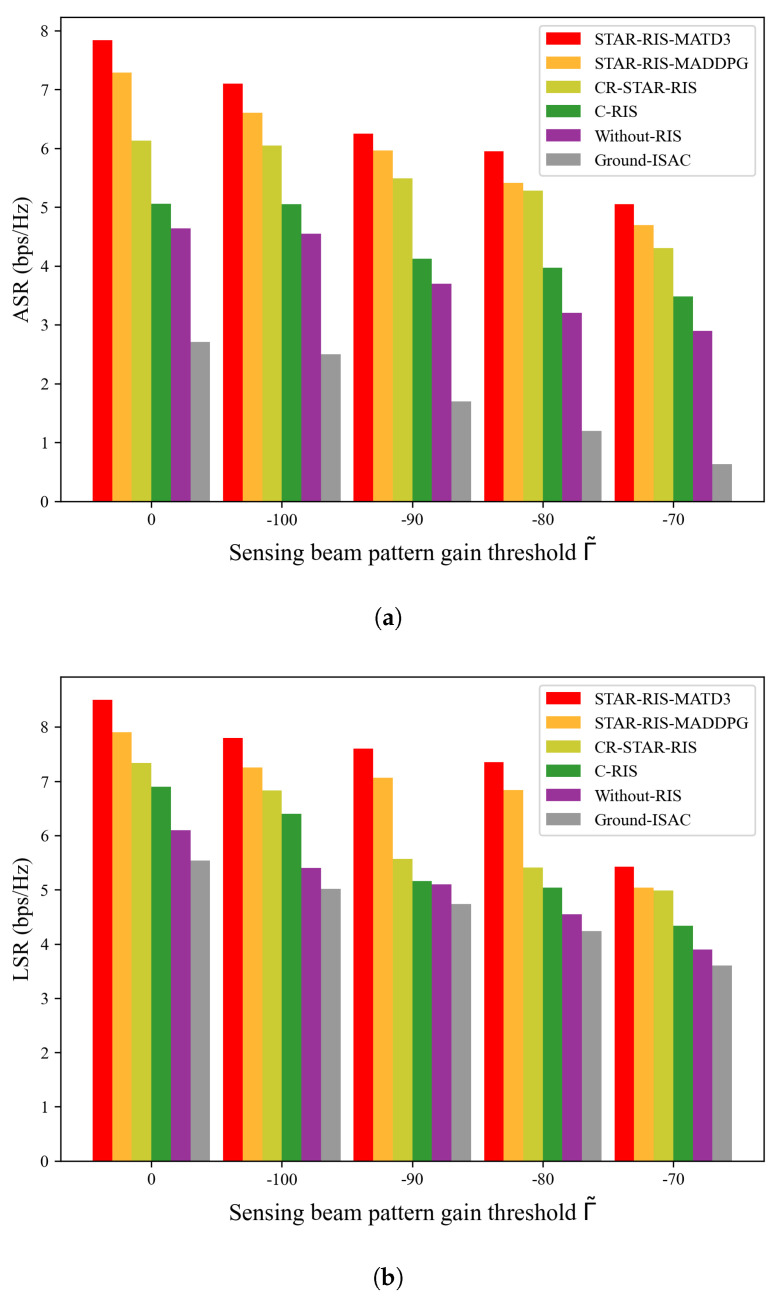
ASR and LSR versus different sensing-beam-pattern gain thresholds $\tilde{\Gamma }$, with $\left(P_{\max},\;M\right)=\left(8\;W,\;15\right)$. (**a**) ASR versus the sensing-beam-pattern gain threshold $\tilde{\Gamma }$. (**b**) LSR versus the sensing-beam-pattern gain threshold $\tilde{\Gamma }$.

Figure 7 illustrates the performance in terms of the ASR and LSR under different sensing-beam-pattern gain thresholds $\tilde{\Gamma }$, with $\left(P_{\max},\;M\right)=\left(8\;W,\;15\right)$. As shown in the figure, a higher sensing-beam-pattern gain threshold resulted in lower ASR and LSR across all algorithms. The apparent reason for this is that, when the sensing-beam-pattern gain threshold $\tilde{\Gamma }$ is higher, a clear trade-off between sensing and communication becomes obvious. The UAV-ISAC allocates more power to the sensing function, which consequently increases the communication burden. The proposed STAR-RIS-MATD3 algorithm consistently outperformed the other algorithms under varying sensing-beam-pattern gain thresholds $\tilde{\Gamma }$, thereby highlighting the superiority of the algorithm proposed in this study.

## 5. Conclusions

This study addressed the security issues associated with the STAR-RIS-assisted UAV-ISAC network and proposed a secure transmission scheme. By introducing the STAR-RIS, the system’s coverage is effectively extended and its network flexibility is enhanced. Specifically, the proposed scheme maximizes the system’s average secrecy rate through jointly optimizing the UAV-ISAC trajectory, the transmit antenna’s beamforming matrix, and the phase and amplitude adjustments of STAR-RIS reflection elements, while meeting communication performance and transmission security constraints. Due to the multi-variable coupling and non-convex optimization, traditional optimization methods are difficult to apply directly in the considered context. To solve this problem, we adopted the MATD3 algorithm, where agents interact with the environment and autonomously learn the optimal strategy. This method can flexibly adapt to dynamic and complex environments, and its effectiveness was verified through simulations. The experimental results demonstrated that the proposed scheme effectively enhances the obtained average secrecy rate, while satisfying communication performance and transmission security constraints, thus providing an effective solution for security optimization in STAR-RIS-assisted UAV-ISAC networks. Future work will focus on integrating multi-antenna reception and incorporating active RIS [25,26] into the STAR-RIS (Active STAR-RIS), which will play a crucial role in enhancing the UAV-ISAC system. This will be a key direction for our future research. Overall, the proposed system and methods demonstrate significant potential for practical applications.

## Figures and Tables

**Table 1:** List of simulation parameters.

Notation	Definition	Value
*H*	UAV-ISAC operating altitude	40–60 m
$N_{t}$	Number of ULA antennas	6
*M*	Number of STAR-RIS reflection elements	15
$P_{\max}$	Maximum power budget	8 W
$\sigma^{2}=\sigma_{k}^{2}=\sigma_{e}^{2}$	AWGN power	−90 dBm
$\tilde{\Gamma }$	Beam pattern gain threshold	−80 dBm
$\Gamma_{e}$	Maximum eavesdropping SINR threshold	−10 dBm
*K*	Number of communication users	4
*E*	Number of sensing users	2

## Data Availability

The data presented in this study are available in Section 4.
